# Comparison of Maternal Serum Magnesium Level in Pre-eclampsia and Normal Pregnant Women

**DOI:** 10.5812/ircmj.10394

**Published:** 2013-12-05

**Authors:** Zohreh Tavana, Sara Hosseinmirzaei

**Affiliations:** 1 Department of Obstetrics and Gynecology, Shiraz University of Medical Sciences, Shiraz, IR Iran

**Keywords:** Pre-Eclampsia, Serum, Magnesium, Pregnancy

## Abstract

**Background:**

Preeclampsia is a pregnancy multisystem disorder of unknown etiology. It is a significant cause of maternal and fetal morbidity and mortality.

**Objectives:**

Due to the significant role of magnesium in physiological regulation of blood pressure, this study was conducted to measure the level of magnesium in pre-eclampsia and control groups since the beginning of the pregnancy.

**Materials and Methods:**

We enrolled 500 pregnant women with gestational age of 18-22 weeks who had referred to the Section of Obstetrics and Gynecology of Hafez hospital of Shiraz. Initially, blood samples were obtained from all subjects. 26 cases with diagnosis of preeclampsia were detected at the next referral. For each case, two normal pregnant women, at the same gestational age, were considered as the control group. The second blood samples were obtained from all the cases and controls. All of the samples were sent to check the level of magnesium. The data was analyzed with the SPSS and Student’s t-test.

**Results:**

The initial level of magnesium in pre-eclampsia women was not only significantly less than the control group (1.81 ± 0.25 mg/dl vs. 2.3 ± 0.44 mg/dl, P < 0.001), but also the secondary level was low, when the diagnosis was confirmed (1.72 ± 0.38 mg/dl vs. 2.2 ± 0.63 mg/dl, P < 0.05).

**Conclusions:**

We found a gradual decrease in mean serum magnesium level with increasing period of gestation in the pre-eclampsia women. This implicates that the level of magnesium in preeclampsia was lower than the control group since the beginning of pregnancy. According to our results, checking the level of magnesium should be considered as the predicting factor of preeclampsia during the first evaluation of pregnancy.

## 1. Background

Preeclampsia is a disorder of widespread vascular endothelial malfunction and vasospasm, which occurs after gestational age of 20 weeks and can present as late as 4-6 weeks postpartum. Preeclampsia is deﬁned as a blood pressure of at least 140/90 mmHg measured on two occasions each 6 h apart, accompanied by proteinuria of at least 300 mg per 24 h, or at least 1+ on dipstick testing. It is clinically defined by hypertension and proteinuria, with or without pathological edema (1). The global incidence of preeclampsia has been estimated at 5-14% of all pregnancies. In developing nations, the incidence of the disease has been reported as 4-18% (2) with hypertensive disorders being the second most common obstetric cause of stillbirths and early neonatal deaths. The pathophysiology of preeclampsia, likely involves both maternal and fetal factors. Abnormalities in the development of placental vasculature early in pregnancy may result in relative placental under perfusion, hypoxia and ischemia which then lead to the release of antiangiogenic factors into the maternal circulation that alter maternal systemic endothelial function and cause hypertension and other manifestations of the disease. However, the molecular basis for placental dysregulation of these pathogenic factors remains unknown, and the role of angiogenic proteins in early placental vascular development are under investigation (3). Perfusion is diminished due to vascular hem concentration and third spacing of intravascular fluids. Preeclampsia is also accompanied by exaggerated inflammatory response and inadequate endothelial activation. Coagulation cascade activation and resultant micro thrombi formation further compromise blood flow to organs (3). However, many clinical studies have shown the relationship between aggravation of hypertension and change in maternal serum level of various minerals during pregnancy (4, 5). Preeclampsia is associated with increased risks of cerebrovascular complications, disseminated intravascular coagulation (DIC), placental abruption and maternal death. Preeclampsia in developing countries has been found to have a great impact because the diet of pregnant mothers are not rich enough with essential minerals and vitamins (6). Inadequate dietary intake is harmful for the mother and the growing fetus. Previous studies have shown a signiﬁcant reduction in the level of serum magnesium in pre-eclampsia pregnant mothers (7). This result agrees with the physiological role of magnesium; magnesium is one of the essential intracellular cautions and an important cofactor for activation of many enzymes. Magnesium has a significant role in pathophysiological regulation of blood pressure because it effects contractility and tone of blood vessels (8). Results from various studies have shown the relationship between level of elements in serum of mothers and Preeclampsia. Previous studies have evaluated the serum magnesium of pregnant women with the diagnosis of preeclampsia, but no one has measured the magnesium level of the pre-eclampsia women since the beginning of their pregnancy.

## 2. Objectives

The aim of this study is to measure serum levels of magnesium in pre-eclampsia and normal pregnancy since the beginning of pregnancy. Each of the participants were evaluated and followed from the beginning of their pregnancy. The main purpose of the present study was to determine whether pregnant women with pre-eclampsia would have lower serum magnesium levels in comparison with members of the control group.

## 3. Materials and Methods

### 3.1. Participants

This cross-sectional study was conducted in the department of Obstetrics and Gynecology at Hafez Hospital of Shiraz University of Medical Science, Shiraz City, Iran. Sample size for 5% maximum error was estimated with the Power SCC software to be 26 (alpha = 0.05, LST = 0.3, SD = 0.33). The incidence of preeclampsia is about 8-10% of pregnancies (9). Research population in the present study included 500 pregnant women between 18-22 weeks of gestational age. Participants included 78 pregnant women in clinical (N = 26) and control (N = 52) groups that were selected by the purposive sampling method. Exclusion criteria for the members of the clinical group were: a history of chronic or transient hypertension, renal or cardiovascular disease, diabetes mellitus or history of consumption of magnesium supplement. The present study was approved by the ethical board of the Shiraz University of Medical Science (number of certificate: 89-01-01-2680 date: 6/06/2011). Informed consent was obtained from each participant before their recruitment. 

### 3.2. Materials

A demographic and pregnancy sheet and a laboratory test were used in this study. The demographic sheet included a few items about name and surname, contact number, age, parity, the gestational age of pregnancy based on LMP or sono 8-12 Week and blood pressure. 5 ml venous blood sample was aspirated from the participants’ antecubital vein. Blood samples were allowed to clot at room temperature and then centrifuged at 1500 rpm for 15 minutes. The samples were sent to the laboratory to check the level of magnesium. Serum magnesium was measured by the colorimetric method and use of Xylidl Blue by an auto-analyzer (Selectra, Netherlands). The coefficient of variance of intra-assay and inter-assay precision of serum magnesium was 0.92% and 1.09%, respectively.

### 3.3. Procedure

After completing the demographic sheet, the second samples of venous blood were obtained from the cases and controls. All of the serum samples were sent to the laboratory of Faghihi hospital to check the level of magnesium. It is important to note that, there was no decrease in sample size during the study. The data was analyzed with the SPSS software package version 15.0 and expressed in terms of mean, standard deviation (SD), and percentage. Continuous variables were compared with the Student’s t-test and paired sample t-test. A P value of < 0.05 was considered to be statistically significant.

## 4. Results

The present study enrolled 500 pregnant women during the first phase of the project. All of them were in the age group of 20-35 years (mean age ± SD = 29.7 ± 5.2), with period of gestation between 18 and 22 weeks (mean ± SD = 19.2 ± 3.5). During the next referral, from all subjects, 26 pregnant women were detected with preeclampsia and were compared with the control group. 52 normal pregnant women, at the same gestational age, were considered as the control group. Average gestational age at the time of the second sample was: 34.21 ± 3.58 for the preclamptic group and 33.4 ± 3.20 for the control group. Demographic and clinical characteristics of the participants in both groups are shown in Table 1. Age, parity and gestational age between pre-eclampsia women and control group are not signiﬁcantly different (P < 0.05). 

**Table 1.** Demographic Data of Study Groups

Variables^a^	Control (n = 52)	Preeclampsia (n = 26)	P value
**Age, y**	27.2 ± 4.5	28.3 ± 4.6	0.31
**Gestational age (weeks)**	34.21 ± 3.58	33.4 ± 3.20	0.33
**Parity, %**			NS
Nulliparous	59.6 (n = 31)	69.2 (n = 18)	
Multiparous	40.4 (n = 21)	30.8 (n = 8)	

^a^ Values are expressed as mean ± SD.

In Table 2, the serum magnesium in pre-eclampsia pregnant women was significantly less than the control group that consisted of normal pregnant women (1.72 ± 0.38 mg/dl vs. 2.2 ± 0.63 mg/dl, P < 0.05). 

**Table 2.** Mean Serum Level of Magnesium in Study Groups

Variable	Control Group	Preeclampsia	P value
**Serum magnesium (mg/dl)**	2.2 ± 0.63	1.72 ± 0.38	0.006

Comparison was also done for serum magnesium level at different weeks of the gestational period. Initial level of magnesium was obtained when the women were at 18-22 weeks of gestational age and secondary level was determined, when the diagnosis of preeclampsia was confirmed (30-35 weeks). Both initial and secondary levels of magnesium of pre-eclampsia women were lower than the control. The serum magnesium decreased as the gestational period increased. This decline was statistically significant (Table 3). 

**Table 3.** Serum Magnesium Levels at Different Gestation Periods

Period of Gestation, week	Serum Magnesium of Control Group (mg/dl)	Serum Magnesium of Pre-eclampsia Women (mg/dl)	P value
**18-22**	2.3 ± 0.44	1.81 ± 0.25	< 0.001
**30-35**	2.2 ± 0.63	1.72 ± 0.38	0.006
**Paired t test**	0.35 (NS)	0.32 (NS)	

## 5. Discussion

Preeclampsia is a syndrome characterized by the onset of hypertension and proteinuria after 20 weeks of gestation and affects approximately 5-18% of pregnancies (1). Preeclampsia is an important disease to screen, as it is common, has significant health outcomes and increases maternal and perinatal mortality. However, although numerous screening tests for preeclampsia have been proposed over the past few decades, no test has so far been able to appropriately screen for the disease and no well-established measurement for initial prevention has been designed (10, 11). Mineral deficiencies like calcium, magnesium, zinc, etc., have been identified to cause significant health problems for women of reproductive age, especially in developing countries due to inadequate dietary intake. The risk of deficiency becomes increased during pregnancy because of increased need of the growing fetus for various nutrients (12). Changes in levels of these elements could affect pregnancy. One of the problems that may be influenced by nutrient deficiencies is preeclampsia. Recently, more emphasis has been laid on the relationship between maternal serum level of elements and occurrence of preeclampsia (13, 14). The present study showed that serum magnesium level was significantly reduced in pre-eclampsia mothers compared with the healthy control group. The level of magnesium in pre-eclampsia women was not only significantly low when the diagnosis was confirmed, but also the initial level from early on in the pregnancy was lower than the control group. These ﬁndings conﬁrmed that hypomagnesaemia may be one of the etiologies of preeclampsia. A study by Seydoux J revealed that serum magnesium decreased with progress in pregnancy (15), but the design and method of our study was different from similar previous studies. This was the first investigation, where serum magnesium level was measured in pre-eclampsia women and was compared with the control group since early on in the pregnancy. We found a gradual decrease in mean serum magnesium level with increasing period of gestation in normotensive women, less than pre-eclampsia women. This suggests that level of magnesium in pre-eclampsia women was lower than the control group since early in the pregnancy.

In conclusion, according to the results from our research, the level of magnesium should be considered as a predicting factor of preeclampsia during the first evaluation of pregnancy. A study by Kulier et al. (16) suggested that the intake of magnesium supplements since the beginning of pregnancy may reduce the rate of preeclampsia among pregnant women. Some studies have reported that serum magnesium in preeclampsia is not diﬀerent from that of normal pregnancy (17, 18). This diﬀerence may be due to the variation in the study population and the dietary intake. Systemically, magnesium lowers blood pressure and alters peripheral vascular resistance. Thus, it can be advantageous for pre-eclampsia women. However, the actual role of magnesium supplements should be further investigated in prevention of preeclampsia.
